# Identification and functional characterization of a novel *surfactant protein A2* mutation (p.N207Y) in a Chinese family with idiopathic pulmonary fibrosis

**DOI:** 10.1002/mgg3.1393

**Published:** 2020-06-30

**Authors:** Lv Liu, Jieli Qin, Ting Guo, Ping Chen, Ruoyun Ouyang, Hong Peng, Hong Luo

**Affiliations:** ^1^ Department of Respiratory Medicine, Diagnosis and Treatment Center of Respiratory Disease The Second Xiangya Hospital of Central South University Changsha Hunan China

**Keywords:** apoptosis, ER stress, idiopathic pulmonary fibrosis, mutation, SFTPA2

## Abstract

**Background:**

Idiopathic pulmonary fibrosis (IPF) is a serious disorder with a high mortality rate worldwide. It is characterized by irreversible scarring of the lung parenchyma resulting from excessive collagen production by proliferating fibroblasts/myofibroblasts. Previous studies have revealed that mutations in surfactant protein‐related genes and telomerase complex genes are crucial underlying genetic factors.

**Methods:**

In this study, we enrolled a family with IPF from the central southern region of China. Whole‐exome sequencing was employed to explore candidate genes in this family. Real‐time PCR and western blotting were used to study the functions of the identified mutations in vitro.

**Results:**

A novel mutation (NM_001098668.4: c.619A>T; NP_001092138.1: p.N207Y) in *surfactant protein A2* (*SFTPA2*,), having not been previously reported to be a mutation, was identified and co‐separated with all affected individuals in the IPF family. Functional research further revealed that the novel mutation affects the secretion of SFTPA2 protein and induces endoplasmic reticulum stress as well as apoptosis in A549 cells.

**Conclusion:**

We are confident that this novel mutation (NM_001098668.4: c.619A>T; NP_001092138.1: p.N207Y) in *SFTPA2* is the genetic mutation of the IPF family. Our study not only confirms the importance of SFTPA2 in IPF but also expands the spectrum of *SFTPA2* mutations and contributes to the genetic diagnosis and counseling of IPF patients.

## Graphical abstract

Mutation in SFTPA2 was rare reported, and we identified a novel pathogenic mutation (p.N207Y) of SFTPA2 gene in idiopathic pulmonary fibrosis (IPF) patients in this study. Functional study further confirmed the novel mutation may affect the expression and secretion of surfactant protein A2 protein and induce endoplasmic reticulum stress and apoptosis. Our study not only confirmed the importance of SFTPA2 in IPF, but also expanded the spectrum of SFTPA2 mutations and contributed to the genetic diagnosis and counseling of IPF patients.

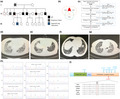

## 1 INTRODUCTION

Idiopathic pulmonary fibrosis (IPF) is a chronic fatal interstitial pulmonary disease characterized by irreversible scarring of the lung parenchyma resulting from excessive collagen production by proliferating fibroblasts/myofibroblasts (Lederer & Martinez, 2018; Martinez et al., 2017). Exercise‐induced breathlessness and chronic dry cough are the prominent symptoms (Martinez et al., 2017). The prevalence of IPF is estimated to be slightly higher in men (20.2/100,000) than in women (13.2/100,000) (Richeldi, Collard, & Jones, 2017). The mean age at presentation is 66 and the average life expectancy of IPF patients is only 3 years from diagnosis to death (King & Nathan, 2017).

To date, more than eight genes divided into two groups underlying IPF have been identified. (I) surfactant protein‐related genes, including *surfactant protein C* (*SFTPC*; OMIM 178620) (Nogee et al., 2002), *surfactant protein A2* (*SFTPA2*; OMIM 178642) (Wang et al., 2009), *surfactant protein A1* (*SFTPA1*; OMIM 178630) (Selman et al., 2003) and *ATP‐binding cassette transporter A3* (*ABCA3*; OMIM 601615) (Shulenin et al., 2004); and (II) telomere‐related genes, such as *telomerase reverse transcriptase* (*TERT*; OMIM 187270) (Armanios et al., 2007), *telomerase RNA component* (*TERC*; OMIM 602322) (Vulliamy et al., 2004), *poly(A)‐specific ribonuclease* (*PARN*; OMIM 604212) (Stuart et al., 2015), and *regulator of telomere elongation helicase 1*(*RTEL1*; OMIM 608833) (Stuart et al., 2015). In addition, the most recent studies indicated that *MUC5B*, *ELMOD2 CSF3R, DSP,* and *LAMA3* present a statistically significant relationship with IPF (Deng et al., 2018). Current large GWAS data also suggest that almost 30% of IPF cases are caused by common genetic mutations (Fingerlin et al., 2013).

In this study, we analyzed an IPF pedigree from the central southern region of China. An autosomal dominant inheritance pattern was identified in this family. Whole‐exome sequencing was employed to detect the pathogenic mutation of the affected individuals.

## 2 METHODS

### 2.1 Ethical compliance

The study was approved by the Ethics Committee of the Second Xiangya Hospital of Central South University and performed in accordance with the principles enshrined in the Declaration of Helsinki. Written informed consent was obtained from the patients.

### 2.2 Study population

**Figure 1:**
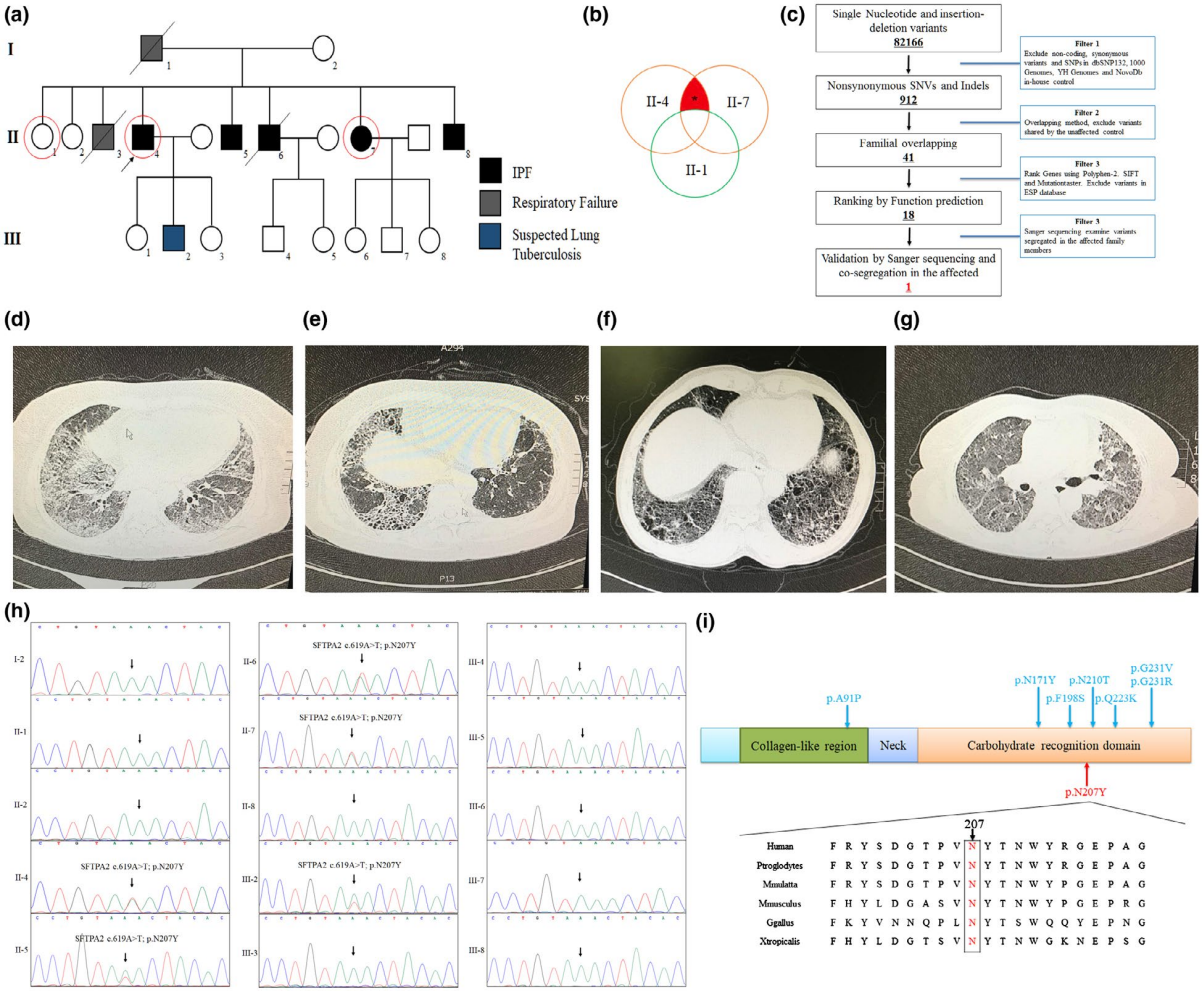
Clinical and genetic information of the family. (a) The clinic and genetic data of an IPF family with *SFTPA2* novel mutation. Squares indicate male family members; circles, female members; close symbols, the patients; open symbols, unaffected members; arrow, proband. (b) Overlapping filter strategy. Asterisks denotes remaining mutations for further analysis that are present in two affected members (II‐4 and II‐7) but not in the normal control (II‐1). (c) Schematic representation of the filter strategies employed in our study. The chest HRCT result of the patient (d) II‐4, (e) II‐5, (f) II‐6, and (g) II‐7. (h) Sanger DNA sequencing chromatogram demonstrates the heterozygosity for a *SFTPA2* mutation (c.619A>T/p.N207Y). (i) Alignment of multiple SFTPA2 protein sequences across species. The N207 affected amino acid locates in the highly conserved amino acid region in different mammals. Letters looped in red show the N207 site, blue letter represent the reported mutations of SFTPA2. HRCT, high‐resolution computed tomography

The Review Board of the Second Xiangya Hospital of Central South University approved this research. All patients gave written informed consent. The clinical data and peripheral blood were collected from the large IPF family (Figure 1a). The final diagnosis of the patients was based on high‐resolution computed tomography (HRCT) and/or transbronchial lung biopsy, after referring to the ATS/ERS/JRS/ALAT guidelines published in 2011, which excluded known causes of interstitial lung disease (ILD) (Raghu et al., 2011). At least two experts in pulmonary disease, two radiologists and rheumatologists independently reviewed each patient's clinical data.

### 2.3 DNA extraction

Genomic DNA was extracted from peripheral blood lymphocytes of all subjects by using the JetFlex™ Genomic DNA Purification Kit (Invitrogen™).

### 2.4 Whole exome sequencing

Whole‐exome sequencing was used to analyze the genetic factors of the large IPF family. The proband (II‐4), one healthy member (II‐1) and an affected member (II‐7) were chosen for the whole exome sequencing at the Novogene Bioinformatics Institute (Figure 1b). Agilent SureSelect Human All Exon V6 kits was undertaken to capture the exomes and the sequencing platform was an Illumina HiSeq X‐10. The strategies for data filtering referred to Figure 1c as our previous described (Liu & Luo, 2018).

### 2.5 Cell culture

The A549 cell line was purchased from the Advanced Research Center of Central South University and maintained at 37°C in a humidified, 5% CO_2_‐controlled atmosphere in medium/RPMI‐160 medium supplemented with 10% fetal bovine serum, 50 IU/ml penicillin, 50 μg/ml streptomycin, and glutamine.

### 2.6 Mutagenesis and cell transfection

The wild‐type *SFTPA2* CDS (NM_001098668) with a C‐terminal HIS‐tag in the pEnter was designed by us. The p.N207Y‐*SFTPA2* missense mutation was constructed into the above vector using the QuikChange Lightning SiteDirected Mutagenesis Kit (Agilent Technologies). Sanger sequencing was applied to check the constructs. A549 cells were transiently transfected with 2 μg *SFTPA2*‐HIS‐pEnter plasmids (WT and/or mutation) using Lipofectamine™ 2000 CD Transfection Reagent (Invitrogen™), according to the manufacturer's instructions and cultured for 72 hr.

### 2.7 Real‐time qPCR and western blot

Real‐time PCR referred to our previous study (Liu & Luo, 2018). For western blotting, one milliliter of cultured medium was removed from each well and centrifuged at 16,000 × *g* for 10 min at 4°C, and cell protein was extracted using RIPA lysis buffer and the concentration was measured using a BCA kit (Thermo Fisher Scientific). Bis‐Tris NuPAGE gels (4%–12%) were used to separate the protein by electrophoresis. Chemiluminescent signals were scanned using a chemiluminescent imaging system (Alpha Innotech). The antibodies against HIS, CHOP, GRP78, Caspase 3, and GAPDH were purchased from Cell Signaling Technology.

## 3 RESULTS

In this study, we enrolled a large family with IPF and other pulmonary diseases (Figure 1a, Table 1). The proband (II‐4), a 63‐year‐old male, showed typical symptoms of cough with little sputum for nearly 4 years. Chest HRCT presented evidence of usual interstitial pneumonitis (UIP) (Figure 1d). Further investigation of the family history revealed that his two brothers (II‐5 and II‐6) and one sister (II‐7) were all diagnosed with IPF according to chest HRCT examination (Figure 1e–g). Both his father (I‐1) and one brother (II‐3) died from respiratory failure according to the description of the proband. In addition, the III‐2 refused to take clinical testing due to far distance from our hospital, but his parents indicated that he (III‐2) suffered from lung tuberculosis according to the diagnosis of another hospital several years ago.

**Table 1:** Clinic data of familial IPF members

Member	Sex	Age	Symptom	Diagnosis	HRCT
I‐1	Male	62 (death)	Cough, dyspnea	Died from respiratory failure	/
II‐3	Male	50 (death)	Cough, dyspnea	Died from respiratory failure	/
II‐4	Male	63	Cough, dyspnea	IPF	UIP
II‐5	Male	58	Dyspnea	IPF	UIP
II‐6	Male	37 (death)	Hemoptysis	IPF	UIP
II‐7	Female	56	Cough, dyspnea	IPF	UIP
III‐2	Male	45	Cough	Suspected lung tuberculosis	/

Abbreviations: HRCT, high‐resolution computed tomography; IPF, idiopathic pulmonary fibrosis; UIP, usual interstitial pneumonia.

Whole‐exome sequencing was undertaken to detect the genetic mutation of the family. Basic whole‐exome sequencing data are presented in Table S1. After data filtering, as shown in Figure 1b,c, we found 18 candidate mutations including the novel mutation (NM_001098668.4: c.619A>T; NP_001092138.1: p.N207Y) of *SFTPA2* (Table 2). Further Sanger sequencing revealed that only the novel mutation of *SFTPA2* (NM_001098668.4: c.619A>T; NP_001092138.1: p.N207Y) was present in the affected individuals (II‐4, II‐5, II‐6, II‐7, and III‐2) and absent in the healthy family members (I‐2, II‐1, II‐2, II‐8, III‐1, III‐4, III‐5, III‐6, III‐7, and III‐8) (Figure 1h). This novel mutation, resulting in the substitution of asparagine with tyrosine at position 207 in exon 6 of the *SFTPA2* gene, was located in a conserved site (Figure 1i).

**Table 2:** Gene list of Sanger sequencing validation and co‐segregation analysis

CHR	POS	REF	ALT	Gene Name	AA Change	SIFT	Polyphen2	MutationTaster
2	11300609	G	A	PQLC3	PQLC3:NM_001282710:exon2:c.G161A:p.R54Q	0.18,T	0.924,D	0.874,D
4	108866135	T	G	CYP2U1	CYP2U1:NM_183075:exon2:c.T500G:p.F167C	0,D	0.499,P	1.000,D
4	185618950	G	T	CENPU	CENPU:NM_024629:exon12:c.C994A:p.P332T	0.26,T	0.995,D	1.000,D
8	93027036	G	A	RUNX1T1	RUNX1T1:NM_175636:exon2:c.C128T:p.T43M	0.16,T	0.796,P	1.000,D
8	145577107	G	A	TMEM249	TMEM249:NM_001252404:exon4:c.C514T:p.R172C	0,D	0.06,B	1.000,D
9	18928178	C	T	FAM154A	SAXO1:NM_001287049:exon4:c.G1102A:p.E368K	0.05,D	0.011,B	1.000,D
10	81317093	T	A	SFTPA2	SFTPA2:NM_001098668:exon6:c.A619T:p.N207Y	0.01,D	0.996,D	0.986,D
10	93390295	C	G	PPP1R3C	PPP1R3C:NM_005398:exon2:c.G343C:p.D115H	0.44,T	0.074,B	1.000,D
11	93844222	C	T	HEPHL1	HEPHL1:NM_001098672:exon18:c.C3199T:p.R1067C	0.04,D	0.717,P	0.953,D
12	56078934	G	A	ITGA7	ITGA7:NM_001144996:exon25:c.C3334T:p.R1112W	0.55,T	0.003,B	1.000,D
12	121437335	C	T	HNF1A	HNF1A:NM_000545:exon9:c.C1673T:p.P558L	0.69,T	0.24,B	1.000,D
17	56540247	C	G	HSF5	HSF5:NM_001080439:exon4:c.G1438C:p.A480P	0.01,D	0.002,B	0.997,N
19	1011184	G	A	TMEM259	TMEM259:NM_001033026:exon10:c.C1228T:p.L410F	0.07,T	0.987,D	1.000,D
19	4254397	C	T	CCDC94	CCDC94:NM_018074:exon4:c.C316T:p.R106W	0,D	0.988,D	0.872,D
20	61526496	G	A	DIDO1	DIDO1:NM_001193369:exon9:c.C2236T:p.R746C	0,D	0.916,D	0.981,N
9	990663	G	GC	DMRT3	DMRT3:NM_021240:exon2:c.1078dupC:p.Q359fs	—	—	1.000,D
15	29415774	CG	C	FAM189A1	FAM189A1:NM_015307:exon11:c.1387delC:p.R463fs	—	—	1.000,D
19	36258936	CA	C	PROSER3	PROSER3:NM_001039887:exon9:c.1190delA:p.Q397fs	—	—	1.000,D

Abbreviations: AB, alternative base identified; B, benign; CHR, Chromosome; D, disease‐causing; N, polymorphism; P, probably damaging; POS, position; RB, reference sequence base; T, tolerated.

**Figure 2:**
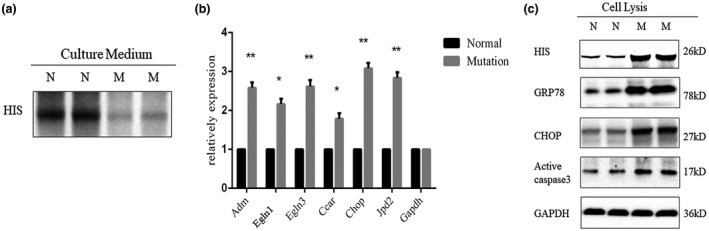
Functional research of novel *SFTPA2* mutation. (a) Western blot analyzed the levels of HIS in normal and mutation cell culture medium with same quality of total protein. (b) Real‐time qPCR analysis the mRNA levels of ER stress related genes and apoptosis related genes. (c) Western blot analysis the levels of HIS, GRP78, BCL2, and GAPDH in normal and mutation cells lysis, ER, endoplasmic reticulum

As previous studies have not reported this transversion (NM_001098668.4: c.619A>T) as a pathogenic mutation, Wt and the p.N207Y mutant plasmids were constructed and transfected into A549 cell lines to perform functional analysis. After culturing for 72 hr, the culture medium, total cell mRNA, and proteins were collected, respectively. Western blot analysis of the expression of His‐SFTPA2 in cell culture medium (the quality of total protein were same in each well) showed that the expression level of Wt was much higher than that of the p.N207Y mutant (Figure 2a), which indicated that the p.N207Y mutation may impair the secretion of SFTPA2. We then performed real‐time PCR to analyze the mRNA levels of ER stress‐related genes (Adrenomedullin, Adm; Prolyl hydroxylase domain 1 and 3, Egln1/3; Jun dimerization protein 2, Jdp2; CHOP) and apoptosis‐related genes (Cell Cycle and Apoptosis Regulatory Protein 1, Ccar1). The results revealed that the expression of endoplasmic reticulum (ER) stress‐related genes and apoptosis‐related genes was both obviously higher in cells harboring the p.N207Y mutation (Figure 2b), suggesting that the mutation (p.N207Y) of *SFTPA2* may induce ER stress and cell apoptosis. Western blot analysis further confirmed this hypothesis (Figure 2c). According to ACMG guidelines, the novel mutation meetings the following criteria from the ACMG guidelines: PS3, PM1, and PM2.

## 4 DISCUSSION

In recent years, an increasing number of studies have discovered that genetic factors play a determinant role in the occurrence and development of IPF in both sporadic and familial cases (Becker, 1989; Spagnolo & Cottin, 2017). It has been proven that up to 20% of people with IPF have another family member with ILD (Fernandez et al., 2012; Garcia‐Sancho et al., 2011). In this study, we employed whole‐exome sequencing to explore the genetic mutation underlying IPF in a Chinese family. A novel mutation (NM_001098668.4: c.619A>T; NP_001092138.1: p.N207Y) in *SFTPA2* was detected in this family. Functional research revealed that this mutation can affect the secretion of the SFTPA2 protein and induce ER stress and apoptosis. Our study is consistent with previous studies showing that pathogenic variations in *SFTPA2* play a critical role in IPF by preventing protein secretion and inducing ER stress (Lawson et al., 2008; Spagnolo & Cottin, 2017; Wang et al., 2009).

*SFTPA2* is one of several genes encoding pulmonary‐surfactant associated proteins. This protein contains three domains: a collagen‐like region, a neck and a carbohydrate‐recognition domain (Silveyra & Floros, 2013; Wang et al., 2009). Previous studies have demonstrated that mutations in the carbohydrate‐recognition domain may result in the formation of an abnormal protein precursor. The abnormal protein accumulates in cells, and ER can cause ER stress (Spagnolo & Cottin, 2017; Wang et al., 2009). Then, ER stress may induce the activation of the unfolded protein response and lead to alveolar epithelial cell apoptosis in cases of long‐standing or severe activation (Chambers & Marciniak, 2014). In our study, the novel p.N207Y mutation was also identified in the carbohydrate‐recognition domain (Figure 1i) and shown to induce ER stress and apoptosis in A549 cells. Our study further confirmed that mutations the in carbohydrate‐recognition domain of SFTPA2 are associated with IPF. To date, only eight *SFTPA2* mutations have been reported in IPF, lung cancer, and ILD patients (van Moorsel et al., 2015; Wang et al., 2009). We have reviewed all the mutations in Figure 1i.

The current methods for the diagnosis of IPF often involve chest HRCT and histology (Chung et al., 2016; Collard, 2017). In addition, all known causes of pulmonary fibrosis need to be excluded, such as connective tissue diseases, chronic hypersensitivity pneumonitis and asbestosis (Martinez & Flaherty, 2017). However, tissue biopsy of IPF patients is not easily to get and phenotypes of IPF patients in HRCT are sundry due to the effect of environmental exposure. Hence, the diagnosis of IPF is somehow difficult to determine. (Spagnolo & Cottin, 2017; Steele & Schwartz, 2013). Genetic sequencing and testing are effective and accurate measures for the diagnosis of IPF patients. Hence, genetic testing can further confirm a clinical diagnosis and allow genetic counseling of families with IPF (Petrovski et al., 2017). In our study, there was one member (III‐2) in whom IPF could not be directly by clinical testing in the family. Our genetic research further confirmed that he was a mutation carrier, and genetic counseling was provided to this family.

In summary, we enrolled a family with IPF to explore the genetic mutation that they harbor by whole‐exome sequencing. A novel mutation of *SFTPA2* (NM_001098668.4: c.619A>T; NP_001092138.1: p.N207Y) was identified in the IPF patients and shown to co‐separate in the affected members. Functional research further confirmed that this mutation can affect the secretion of the SFTPA2 protein and induce ER stress and apoptosis in A549 cells. Our study not only expands the spectrum of *SFTPA2* mutations and contributes to the genetic diagnosis and counseling of IPF patients but also provides a valuable, population‐specific *SFTPA2* mutation that may contribute to further mechanistic and therapeutic research.

## CONFLICT OF INTERESTS

The authors declare that they have no competing interests.

## AUTHOR CONTRIBUTIONS

Dr. Lv Liu performed the genetic analysis and molecular biology experiments including western blot and real‐time PCR; Miss Jieli Qin conducted cell culture and cell transfection; Dr. Ting Guo enrolled clinical data of the family; Dr. Ping Chen, Dr. Ruoyun Ouyang and Dr. Hong Peng enrolled the samples and confirmed the diagnosis; Dr. Hong Luo and Dr. Lv Luo wrote and revised the manuscript. Dr. Hong Luo designed and supported the project. All authors approved the final manuscript.

## Supporting information

Table S1

## Data Availability

The datasets used and/or analyzed during the current study are available from the corresponding author upon reasonable request.
