# Supplementary material for: Identification and functional characterization of a novel surfactant protein A2 mutation (p.N207Y) in a Chinese family with idiopathic pulmonary fibrosis
Source: Mol Genet Genomic Med. 2020 Jun 30;8(9):e1393. doi: 10.1002/mgg3.1393 (PMC7507553; doi:10.1002/mgg3.1393)
Supplement: Supplementary file 1 — Table S1 [file MGG3-8-e1393-s001.docx]

Table S1. Summary of SNPs for exome captured samples and filter procedure

| Categories | II-4 | II-7 | II-1 |
| --- | --- | --- | --- |
| Exome Capture Statistics | | | |
| Target region (bp) | 50226211 | 50323297 | 50334227 |
| Raw reads | 50787614 | 44195860 | 46732670 |
| Raw data yield (Mb) | 9174.51 | 8990.99 | 8873.44 |
| Reads mapped to genome | 50730623 | 44147554 | 46682687 |
| Reads mapped to target region | 50195130 | 43863492 | 46378726 |
| Data mapped to target region (Mb) | 5008.29 | 4242.75 | 4511.01 |
| Coverage of target region (%) | 99.70% | 99.80% | 99.80% |
| Average read length (bp) | 80.51 | 80.50 | 80.5 |
| Fraction of target covered>=10X (%) | 98.80% | 99.00% | 99.00% |
| Fraction of target covered>=20X (%) | 96.70% | 95.80% | 96.10% |
| Fraction of target covered>=30X (%) | 84.10% | 83.80% | 84.10% |
| SNPs for exome capture | | | |
| No. high-confidence genotypes | 113441529 | 113276219 | 11417200 |
| Total number of SNPs | 82166 | 76829 | 78101 |
| Missense | 11060 | 9958 | 10001 |
| Nonsense | 75 | 79 | 81 |
| Splice site | 2639 | 2434 | 2381 |
| Synonymous-coding | 12176 | 11506 | 11876 |
| Hom | 38997 | 37843 | 34851 |
| Het | 43169 | 38986 | 43250 |
| Novel variants | | | |
| Number not in dbSNP | 1248 | 1170 | 1258 |
| Number not in 1000 Genome | 977 | 998 | 1002 |
